# Epidemiologic Insights on Polyarthritis in Dogs in Primary Care Populations

**DOI:** 10.1111/jvim.70213

**Published:** 2025-08-27

**Authors:** Momoko Narita, Takashi Hirano, Eiji Naito, Hiroto Taira, Shunya Yokota, Takanori Inden, Masashi Yuki

**Affiliations:** ^1^ Yuki Animal Hospital Nagoya Aichi Japan

**Keywords:** C‐reactive protein, dog, immune‐mediated polyarthritis, joint, reactive polyarthritis

## Abstract

**Background:**

Polyarthritis (PA) is an inflammatory joint disease, sometimes with vague clinical signs.

**Hypothesis/Objective:**

Examine the occurrence of PA in dogs with increased plasma C‐reactive protein (CRP) concentrations, gait‐related clinical signs, or both, and characterize the epidemiological, clinical, and laboratory findings in dogs with PA.

**Animals:**

Eighty‐one dogs with increased plasma CRP concentrations, gait‐related clinical signs, or both.

**Methods:**

Single institution, prospective study. The occurrence of PA was examined in dogs with increased plasma CRP concentrations, showing gait‐related clinical signs, or both. Information such as breed, age at diagnosis, sex, body weight, clinical signs, laboratory results, clinical diagnosis, treatment, and therapeutic response was determined for dogs with and without PA.

**Results:**

Of 81 dogs, 20 (25%) were diagnosed with PA, representing 11 breeds. Sex distribution was 10 males and 10 females, with a median body weight of 5.4 kg and a median age of 13.8 years at diagnosis. Half of the cases exhibited gait‐related clinical signs. Thirteen dogs had reactive PA, seven had primary immune‐mediated PA, and none had infectious causes. The PA group had higher serum total calcium concentrations than the non‐PA group, and the recovery rate for PA was 90%.

**Conclusions and Clinical Importance:**

Polyarthritis should be included in the differential diagnosis for all dogs with increased CRP concentrations, even those without gait‐related clinical signs. Synovial fluid testing is indicated for dogs with high CRP concentrations, even after treatment for underlying diseases, particularly if they are only displaying vague clinical signs.

**Abbreviations**
CRP: C‐reactive protein
IMPA: immune‐mediated polyarthritis
PA: polyarthritis
RePA: reactive polyarthritis
SLE: systemic lupus erythematosus

## 1 Introduction

Polyarthritis (PA) is an inflammatory joint disease that impacts more than two joints [1]. Dogs with PA exhibit various clinical signs, such as hesitation to move, spontaneous vocalization, stiffness, lameness, and difficulty standing or walking [2, 3]. Sometimes, only vague clinical manifestations are seen, such as fever, loss of appetite, and weight loss [2, 4]. These vague clinical signs can make the diagnosis of PA difficult.

Clinical tests, including physical examination, blood tests, radiography, and synovial fluid analysis are necessary to verify the diagnosis. C‐reactive protein (CRP) is an acute‐phase protein that is increased during inflammation or tissue damage [5]. Increased CRP concentrations serve as a noninvasive biomarker for diagnosing and monitoring idiopathic immune‐mediated polyarthritis (IMPA) because most affected dogs have increased concentrations [6, 7, 8].

Polyarthritis is categorized as infectious or noninfectious (immune‐mediated) based on its cause. Primary IMPA is categorized as erosive and nonerosive types based on radiographic and histologic characteristics. The nonerosive form is most prevalent and can be further classified as PA associated with systemic lupus erythematosus (SLE), breed‐associated PA, or idiopathic IMPA [1, 9]. Few studies have examined the epidemiological features of PA [2, 4, 9].

We aimed to prospectively evaluate the occurrence of PA in dogs with high plasma CRP concentrations, gait‐related clinical signs, or both and to identify the epidemiologic, clinical, and laboratory characteristics in dogs diagnosed with PA. We hypothesized that PA often is missed because of its diverse clinical manifestations.

## 2 Materials and Methods

### 2.1 Case Selection and Experimental Design

The selection process was carried out at Yuki Animal Hospital, a primary care animal hospital, between April 2022 and April 2024. We prospectively included cases that underwent several screening tests: CBC, serum biochemistry profile, urinalysis, thoracic and abdominal radiography, and abdominal ultrasound examination. Only dogs showing clear clinical signs of PA (e.g., reluctance to move, spontaneous vocalization, stiffness, lameness, inability to stand or walk) with or without high CRP concentrations were included. We defined a CRP concentration > 4 mg/dL as high, considering inter‐ and intraindividual variability [10]. The included dogs had synovial fluid samples collected by arthrocentesis. Data gathered included breed, age at diagnosis, sex, weight, clinical signs, and vaccination dates. Dogs exhibiting severe respiratory signs or bleeding tendency were excluded because their participation could lead to respiratory distress or anemia.

### 2.2 Blood Testing

Blood samples for CBC and serum or plasma analysis were collected from all dogs by venipuncture. Samples were placed in tubes with or without anticoagulants. An automated hematology analyzer was used for the CBC (ProCyte Dx, IDEXX Laboratories, Tokyo, Japan or Celltac *α*, Nihon Kohden, Tokyo, Japan). Serum or plasma was separated from blood samples within 30 min of collection, and all samples were analyzed on the same day they were collected.

The serum biochemical profile including CRP concentration was measured using a dry chemistry analyzer (FUJI DRI‐CHEM 7000 V, FUJIFILM Corporation, Tokyo, Japan). Samples were sent to a commercial laboratory if in‐hospital testing results were inconclusive.

### 2.3 Arthrocentesis

Synovial fluid was collected from each dog by arthrocentesis from at least three joints, primarily the carpus, tarsus, and stifle. Direct cytologic examination of the synovial fluid was conducted after sample collection using Diff‐Quik‐stained smears. A diagnosis of PA was made when cytologic analysis indicated suppurative inflammation in at least two joints. Suppurative inflammation in the synovial fluid was defined as > 2 nucleated cells per field at 400× magnification and neutrophils comprising > 12% of the nucleated cells [1, 11].

### 2.4 Diagnostic Imaging

Thoracic and abdominal radiography (at least two orthogonal views) and abdominal ultrasound examination were performed to evaluate underlying causes. Dogs diagnosed with PA underwent radiography of the affected joints.

### 2.5 Diagnosis

Dogs were categorized into PA and non‐PA groups based on arthrocentesis results. Dogs in the PA group were classified as: (1) infectious, (2) secondary to a distant immunogenic stimulus (reactive), and (3) primary IMPA (erosive or nonerosive). Infectious arthritis was assessed by cytologic evaluation of synovial fluid samples. Erosive PA was confirmed by radiographic signs of bone lysis within a joint [12]. When dogs with PA had underlying diseases, they were diagnosed with reactive IMPA. Reactive IMPA has been linked to several antigenic stimuli [1]. The underlying causes were classified as: (1) infectious diseases distant from the joints, (2) gastrointestinal or hepatic diseases, (3) neoplasia distant from the joints, and (4) drugs or vaccinations [1]. Drugs and vaccinations were considered to be likely the cause if given before clinical signs appeared, if clinical manifestations emerged within a month of exposure, and if no other underlying cause of PA could be identified [9]. Dogs with noninfectious, nonerosive PA without underlying diseases were diagnosed with primary IMPA. Primary IMPA is categorized into breed‐associated PA, PA associated with SLE, and idiopathic IMPA. Breed‐associated PA was diagnosed when primary IMPA was diagnosed in young dogs of particular breeds [13]. The diagnosis of PA associated with SLE was made using established criteria [14]. Primary IMPA failing to meet the criteria for breed‐associated PA and PA associated with SLE was classified as idiopathic IMPA. Dogs in the non‐PA group were classified according to the causes of increased CRP concentrations, showing gait‐related clinical signs or both: (1) infectious diseases, (2) gastrointestinal or hepatic diseases, (3) neoplasia, (4) drugs or vaccinations, (5) others.

### 2.6 Therapeutic Interventions and Outcomes

Dogs initially were treated for the underlying diseases that may have been present. Prednisolone and immunosuppressants were administered at the discretion of the clinician. Remission was defined as either resolution of suppurative synovial fluid inflammation or normalization of CRP concentration and absence of clinical signs including lameness [7].

### 2.7 Statistical Analysis

We used a Mann–Whitney *U* test to compare the PA and non‐PA groups for age at diagnosis, weight, CBC, and serum biochemistry profile. We conducted logistic regression analysis for variables showing significant differences in the univariate analysis. *p*‐values < 0.05 were deemed significant. Data were imported into the statistical software R for analysis [15].

## 3 Results

### 3.1 Animals

**FIGURE 1:**
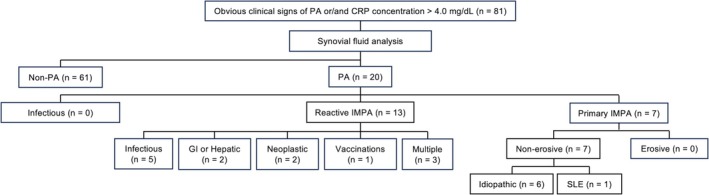
Outline of case selection and classification process. This flowchart illustrates the systematic approach used to include and exclude cases in the study, detailing each step in classifying cases. CRP, C‐reactive protein; GI, gastrointestinal; IMPA, immune‐mediated polyarthritis; PA, polyarthritis; SLE, systemic lupus erythematosus.

**FIGURE 2:**
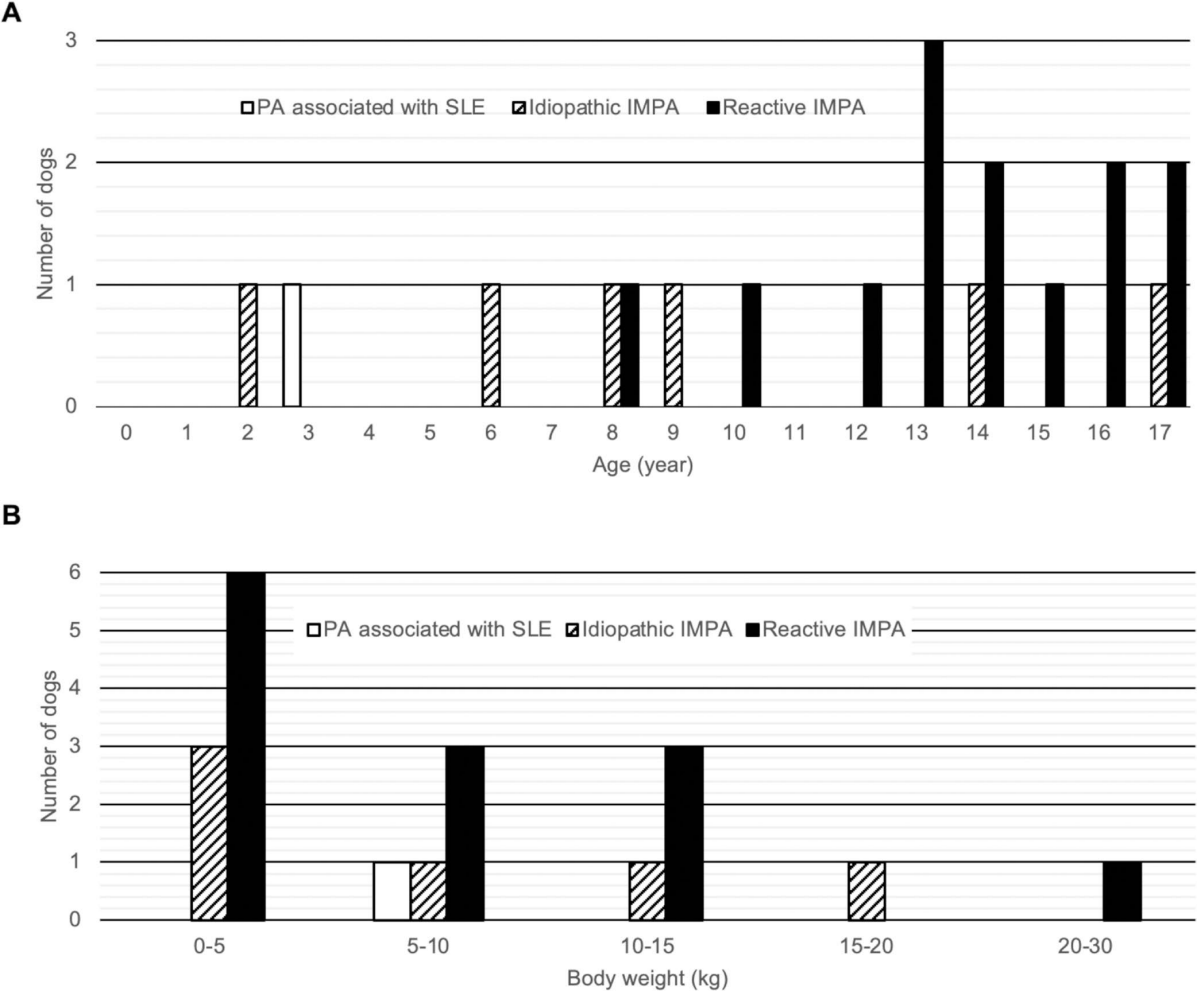
Age and weight distribution of dogs with polyarthritis (PA) types. (A) Age distribution of dogs diagnosed with PA, categorized by type: PA associated with systemic lupus erythematosus (SLE) is represented by clear solid bars, idiopathic immune‐mediated polyarthritis (IMPA) by striped bars, and reactive IMPA by black solid bars. (B) Weight distribution of the same groups of dogs with PA, using the same color coding as above.

Eighty‐one dogs were included, of which 78 had increased CRP concentrations and 12 had lameness. Twenty dogs (25%) were diagnosed with PA (Figure 1). The PA group comprised 10 males (4 intact, 6 neutered) and 10 females (3 intact, 7 spayed). The median age of dogs with PA was 13.8 years (range, 2.2–17.9 years; Figure 2A). Median body weight was 5.4 kg (range, 2.0–29.6 kg; Figure 2B). Eleven breeds were identified: Miniature Dachshund (*n* = 5), Chihuahua (*n* = 3), Toy Poodle (*n* = 3), Pembroke Welsh Corgi (*n* = 2), Siberian Husky (*n* = 1), Italian Greyhound (*n* = 1), Shetland Sheepdog (*n* = 1), Pomeranian (*n* = 1), French Bulldog (*n* = 1), Papillon (*n* = 1), and mixed breed (*n* = 1).

### 3.2 Diagnosis and Investigation

Seven dogs were diagnosed with primary IMPA, 13 dogs had reactive IMPA, and there were no cases with infectious causes (Figure 1). Among the seven dogs diagnosed with primary IMPA, six had idiopathic IMPA, whereas one had PA associated with SLE. No dog had erosive changes. Among the 13 dogs with reactive IMPA, five had infectious diseases unrelated to the joints (pyometra, *n* = 2; bacterial cystitis, *n* = 1; bacterial prostatitis, *n* = 1; bacterial pyelonephritis, *n* = 1), two had gastrointestinal or hepatic disease (cholecystitis, *n* = 2), two had neoplasia distant from the joints (mammary gland tumors, *n* = 2), three had multiple diseases not related to the joints (acute pancreatitis and bacterial cystitis, *n* = 1; acute hepatitis and bacterial cystitis, *n* = 1; urinary tract tumor and stomach tumor, *n* = 1), and one dog had a vaccination suspected to be contributing to reactive IMPA. Vaccination times were recorded for seven dogs. One of these dogs was vaccinated a month earlier and developed lameness 5 days later. This dog previously had shown lameness after vaccination and was diagnosed with vaccination‐induced reactive IMPA. The underlying diseases of the 81 dogs are summarized in Table 1; neoplasia and infectious diseases comprised a high proportion of the underlying diseases causing reactive IMPA.

**TABLE 1:** Number of dogs with reactive immune‐mediated polyarthritis (IMPA) and non‐PA in each diagnosis.

Diagnosis	Number of reactive IMPA	Number of non‐PA
Infectious diseases	7^a^	17^a^
Gastrointestinal or hepatic diseases	4^a^	29^a^
Neoplasia	3^a^	4
Drugs or vaccinations	1	0
Others	0	15

^a^ Dogs with multiple diseases were included.

Table 2 presents the clinical signs and physical examination findings of dogs with and without PA. In the PA group, 10 dogs (50%) showed lameness, with 3 dogs experiencing lameness in one limb and 7 dogs experiencing lameness affecting multiple limbs. Overall, lameness appeared less prevalent in the non‐PA group than in the PA group, but no obvious differences were noted for other clinical signs and physical examination findings.

**TABLE 2:** Frequency of clinical signs and physical examination findings in dogs with and without polyarthritis (PA).

	Non‐PA (%)	Primary IMPA (%)	Reactive IMPA (%)
	*n* = 61	*n* = 7	*n* = 13
Lameness	3	86	31
Joint pain	2	86	31
Joint swelling	0	0	0
Anorexia	84	29	62
Lethargy	80	29	54
Pyrexia (> 39.2°C)	15	29	8
Weight loss	2	0	0
Gastrointestinal signs	56	0	23
Respiratory signs	8	0	0
Urinary signs	3	0	15

Abbreviations: IMPA, immune‐mediated polyarthritis; SLE, systemic lupus erythematosus.

In dogs with PA, the most frequently observed hematologic abnormality was leukocytosis (10/20, 50%), followed by anemia (8/20, 40%), thrombocytopenia (3/20, 15%), and leukopenia (1/20, 5%). No hematologic abnormalities were found in six dogs (30%). Serum biochemistry profiles had abnormalities such as increased alkaline phosphatase activity (13/19, 68%), increased alanine aminotransferase activity (9/16, 56%), hypoalbuminemia (2/18, 11%), azotemia (6/18, 33%), and hypercalcemia (3/18, 17%).

The Mann–Whitney *U* tests indicated differences in serum total calcium and potassium concentrations between the PA and non‐PA groups (*p* = 0.02 for calcium, *p* = 0.04 for potassium; Table 3). Notably, three dogs in the PA group had hypercalcemia. One dog had hyperkalemia. Logistic regression analysis was conducted on these concentrations. Results indicated that the PA group had a higher serum total calcium concentration than the non‐PA group (*p* = 0.03).

**TABLE 3:** Comparative analysis of age, body weight, CBC, and serum biochemistry profiles in dogs with and without PA.

	No.	Non‐PA	No.	PA	*p* value
		Median (IQR)		Median (IQR)	
Age (years)	61	13.0 (9.0–15.2)	20	13.2 (8.8–15.4)	0.45
Body weight (kg)	61	4.9 (3.2–7.5)	20	5.4 (4.2–11.3)	0.13
White blood cells (×10^3^cells/μL)	61	18 (10–24)	20	16 (12–28)	0.99
Hematocrit (%)	61	43.3 (33.3–47.2)	20	39.8 (34.5–47.7)	0.92
Platelets (×10^4^/μL)	61	38.4 (25.9–50.0)	20	32.7 (23.9–43.0)	0.33
Albumin (g/dL)	53	3.1 (2.7–3.6)	18	3.2 (2.9–3.4)	0.92
Blood glucose (mg/dL)	59	112 (95–128)	17	110 (104–125)	0.93
Alanine aminotransferase (U/L)	61	71 (39–199)	19	69 (41–162)	0.66
Alkaline phosphatase (U/L)	52	248 (97–671)	16	337 (215–644)	0.58
Creatinine (mg/dL)	56	0.68 (0.49–1.18)	17	0.83 (0.43–2.17)	0.54
Blood urea nitrogen (mg/dL)	60	19.2 (12.2–42.2)	18	22.1 (14.5–93.1)	0.54
Calcium (mg/dL)	52	10.9 (9.9–11.4)	18	11.5 (10.9–12.1)	0.02*
Sodium (mEq/L)	59	146 (143–149)	15	146 (145–151)	0.22
Chloride (mEq/L)	59	107 (101–111)	15	112 (105–115)	0.20
Potassium (mEq/L)	59	4.0 (3.8–4.4)	15	4.3 (4.2–4.6)	0.04*
C‐reactive protein (mg/dL)	61	15.1 (7.9–18.6)	20	16.2 (9.9–19.0)	0.76

*Note:* Variables indicated with *represent those for which *p* value was < 0.05.

Abbreviations: IQR, interquartile range; PA, polyarthritis.

Two of 20 dogs with PA had CRP concentrations within the reference interval. These dogs were already receiving treatment with corticosteroids or immunosuppressants for their diagnoses: inflammatory bowel disease (prednisolone, 0.3 mg/kg q48h) or SLE (prednisolone, 0.7 mg/kg/day and chlorambucil, 0.4 mg/m^2^/day). Additionally, two other dogs were treated with corticosteroids or immunosuppressants for chronic hepatitis (prednisolone, 0.28 mg/kg/day and chlorambucil, 2.4 mg/m^2^ q48h) and inflammatory bowel disease (prednisolone, 1 mg/kg q48h and cyclosporine, 8 mg/kg daily).

### 3.3 Therapeutic Interventions and Outcome

**FIGURE 3:**
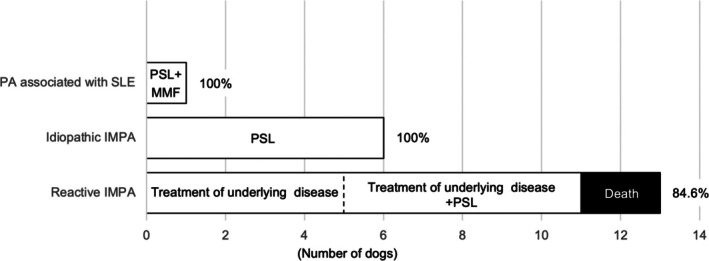
Comparative treatment and recovery rates for various types of polyarthritis. This figure illustrates the treatment and recovery rates for polyarthritis (PA) associated with systemic lupus erythematosus (SLE), idiopathic immune‐mediated polyarthritis (IMPA), and reactive IMPA. Each category is analyzed to highlight differences in therapeutic effectiveness and patient outcomes, providing insights into the management of each condition. Percentages refer to the proportion of dogs that achieved remission. MMF, mycophenolate mofetil; PSL, prednisolone.

The recovery rate for PA was 90%. Dogs with improved cytology or normalization of CRP concentrations had improvement in clinical signs, including lameness. All dogs diagnosed with idiopathic IMPA and treated solely with prednisolone (1.2–2.0 mg/kg/day) showed improvement. A dog with PA associated with SLE, treated with prednisolone (2.0 mg/kg/day) and mycophenolate mofetil (20 mg/kg q12h), also improved. Eleven dogs (84.6%) with reactive IMPA showed improvement. Five of these 11 dogs improved after treatments focused only on their underlying diseases. Six dogs needed additional treatment with prednisolone (2.0 mg/kg/day). Unfortunately, two dogs with reactive IMPA died because of their underlying conditions (Figure 3).

## 4 Discussion

We hypothesized that PA frequently goes unnoticed in dogs with nonspecific clinical signs. Our results indicated that 50% of the dogs with PA had lameness, a smaller population than the 81% noted in a previous study [9]. This finding might indicate that PA featuring only nonspecific signs may be underdiagnosed. Idiopathic IMPA typically is described as the most prevalent type of PA in dogs [2, 9]. In our study, reactive IMPA was the most common type of PA. Dogs with reactive IMPA were more likely to exhibit only nonspecific signs. Earlier research on PA has focused primarily on secondary hospitals [2, 4, 9]. Some dogs with reactive IMPA achieve remission only by treating the underlying condition [1]. These dogs may be underdiagnosed because they have improved before being referred to secondary hospitals.

Evidence suggests that medium to large‐breed dogs are more likely to have noninfectious PA [9], but some studies also have shown that nonerosive PA is more common in small to medium‐sized dogs [2, 4]. The Cocker Spaniel, Labrador Retriever, and American Eskimo breeds are strongly represented [2, 4]. Most of our study dogs were small breeds, such as Miniature Dachshunds, Chihuahuas, and Toy Poodles. This trend might result from a predisposition of small breed dogs to PA or the breed distribution in Japan, which includes many small‐breed dogs [16]. As reported in previous studies [2, 3, 9], we observed no sex‐specific predisposition. Earlier research indicates that most dogs diagnosed with PA were 3–7 years old [2, 4, 9]. The dogs in our study were diagnosed at older ages than those in earlier research. Additionally, our study included a larger proportion of dogs with reactive IMPA, commonly found in older dogs, than in previous studies [2, 9].

The clinical and laboratory findings of our study did not allow for a clear distinction between PA and non‐PA groups. The occurrence of anorexia and lethargy in dogs with PA was similar to earlier reports [2, 6, 17]. The occurrence of pyrexia in reactive IMPA was lower than that in primary IMPA, as observed in previous reports (18%–71%) [2, 4, 6, 17]. Pyrexia may occur less frequently in dogs with reactive IMPA, depending on the nature of the underlying disease. Nevertheless, additional studies with larger case numbers are necessary. Earlier reports have noted gastrointestinal signs in dogs with idiopathic IMPA [1, 2, 9], but no dogs with idiopathic IMPA in our study had gastrointestinal signs. Dogs with reactive IMPA showed gastrointestinal and urinary manifestations linked to their underlying diseases. Swelling in the joints was not noted in our study, possibly because of the inclusion of many small dogs, making it difficult to detect swelling. Prior reports indicated that the laboratory abnormalities associated with IMPA include leukocytosis, nonregenerative anemia, increased alkaline phosphatase activities, and hypoalbuminemia [2]. In our study, some dogs with PA had abnormalities, but these were not substantially different from those in the non‐PA group. These irregularities might be related to the underlying diseases. Univariate analysis showed that serum potassium concentration was higher in the PA group than in the non‐PA group; this difference was not identified in multivariable analysis. No reports link PA to hyperkalemia in human or veterinary medicine. Increasing sample size may be necessary to further assess any potential connection between PA and hyperkalemia. Additionally, serum total calcium concentration was higher in the PA group than in the non‐PA group. In humans, increased alpha‐1‐hydroxylase production by macrophages has been documented in inflammatory joint disease [18]. Alpha‐1‐hydroxylase transforms 25‐hydroxycholecalciferol into its active form, 1,25‐dihydroxycholecalciferol, which increases blood calcium concentrations. A dog experiencing hypercalcemia as a result of idiopathic IMPA has been reported [19]. The dog had increased concentrations of 1,25‐dihydroxycholecalciferol. Although PA can induce hypercalcemia in dogs, only three of our dogs had increased serum total calcium concentrations. A study that includes measuring serum ionized calcium concentration would be necessary to explore a link between PA and hypercalcemia.

In a prior study, treatment benefited 95% of dogs with idiopathic IMPA [17]. In our study, dogs with idiopathic IMPA responded positively to treatment with corticosteroids. Predicting the prognosis of SLE in dogs is challenging and varies considerably [14]. The single dog in our study that exhibited PA associated with SLE showed improvement with treatment using prednisolone and mycophenolate mofetil. Few case reports describe treatment of reactive IMPA [20, 21]. In these reports, reactive IMPA improved with treatment of the underlying infectious disease or neoplasia. However, the ideal treatment for reactive IMPA remains uncertain. We believe that treating the underlying condition should be the primary approach for several reasons: (1) almost 40% of dogs with reactive IMPA improved when the underlying disease was treated; (2) inadequate management of the underlying disease contributed to treatment failure; and (3) the underlying disease frequently was infectious in nature. Immunosuppressive treatment should be considered if there is no improvement after treatment of the underlying disease.

We found that infectious diseases and gastrointestinal or hepatic conditions were more frequently identified as the underlying diseases in reactive IMPA compared with other diseases. A similar finding was reported in a prior study [2]. The frequency of reactive IMPA was notably higher in dogs with infectious diseases and neoplasia. In dogs with infectious diseases or neoplasia, reactive IMPA may need to be considered. In human medicine, microbes responsible for triggering reactive IMPA and predisposing genetic factors associated with reactive IMPA associated with infectious diseases are recognized [22]. The specific microbes and contributing factors in dogs remain unidentified, necessitating additional research. Paraneoplastic arthritides are recognized in humans and occur with numerous solid tumors and hematologic malignancies [23]. Lung adenocarcinoma is the most prevalent, followed by hematologic malignancies and urinary tract cancer [24]. The types of neoplasia responsible for reactive IMPA in dogs remain unclear. We identified mammary gland, urinary tract, and stomach tumors as potential causes of reactive IMPA. Additional research is necessary to determine which types of neoplasia will likely lead to reactive IMPA.

Our study had several limitations. First, synovial fluid tests were conducted on dogs with increased CRP concentrations, gait‐related clinical signs, or both. Reports indicated that increased CRP concentrations were noted in most cases of idiopathic IMPA. Nevertheless, it remains unclear if CRP concentrations increase in other forms of PA. Dogs without gait‐related clinical signs or high CRP concentrations may have been missed. Additionally, two dogs were diagnosed with PA even though their CRP concentrations were normal. These dogs received corticosteroids or immunosuppressants for inflammatory bowel disease or SLE. In cases in which a dog is treated with anti‐inflammatory medications or immunosuppressants, synovial fluid analysis and cytology should be conducted for any lameness, regardless of CRP concentration. Furthermore, when infectious PA is suspected, the synovial fluid should be cultured for microbes. In our study, only direct cytologic examination of the synovial fluid was performed, without microbial culture. Many dogs improved with immunosuppressive treatment. The favorable response to these treatments suggests that infectious PA might not have been represented in our study. However, infectious PA cannot be ruled out in dogs that do not respond to immunosuppressive treatment or in those receiving antibiotics for underlying conditions. Lastly, some dogs were excluded from the study because of severe respiratory signs or bleeding tendencies, potentially biasing case inclusion. Reports indicate that bacterial pneumonia may be an underlying condition for reactive IMPA [2]. We used two automated hematology analyzers to assess CBCs. The ProCyte Dx and an impedance‐based analyzer (similar to Celltac *α*) demonstrated strong correlation in prior research [24].

In conclusion, we demonstrated that even dogs without lameness sometimes had PA. Based on clinical and laboratory findings, it was difficult to discriminate between dogs with PA and those in the non‐PA group. Dogs with reactive IMPA more often exhibited only nonspecific signs than did dogs with other forms of PA. Consequently, synovial fluid analysis is suggested for dogs with high CRP concentrations despite treatment for the underlying disease, even if they only exhibit vague clinical signs.

## Disclosure

Authors declare no off‐label use of antimicrobials.

## Ethics Statement

Approved by the hospital board of the Yuki Animal Hospital (approved on 1 March 2022). All clients gave their informed consent. Authors declare human ethics approval was not needed.

## Conflicts of Interest

The authors declare no conflicts of interest.
